# Characterization of the Drug Resistance Profiles of Patients Infected with CRF07_BC Using Phenotypic Assay and Ultra-Deep Pyrosequencing

**DOI:** 10.1371/journal.pone.0170420

**Published:** 2017-01-20

**Authors:** Szu-Wei Huang, Wei-You Li, Wen-Hung Wang, Yu-Ting Lin, Chih-Hung Chou, Marcelo Chen, Hsien-Da Huang, Yen-Hsu Chen, Po-Liang Lu, Sheng-Fan Wang, Shinichi Oka, Yi-Ming Arthur Chen

**Affiliations:** 1 Center for Infectious Disease and Cancer Research (CICAR), Kaohsiung Medical University, Kaohsiung, Taiwan; 2 Graduate Institute of Medicine, Kaohsiung Medical University, Kaohsiung, Taiwan; 3 Departments of Internal Medicine, Kaohsiung Medical University Hospital, Kaohsiung, Taiwan; 4 Institute of Bioinformatics and Systems Biology, National Chiao Tung University, Hsinchu, Taiwan; 5 Department of Urology, Mackay Memorial Hospital, Taipei, Taiwan; 6 Department of Cosmetic Applications and Management, Mackay Junior College of Medicine, Nursing and Management, Taipei, Taiwan; 7 School of Medicine, Mackay Medical College, New Taipei City, Taiwan; 8 Department of Biological Science and Technology, National Chiao Tung University, Hsinchu, Taiwan; 9 Division of Infectious Diseases, Department of Internal Medicine, Kaohsiung Medical University Hospital, Kaohsiung, Taiwan; 10 Department of Laboratory Medicine, and Department of Internal Medicine, Kaohsiung Medical University Hospital, Kaohsiung Medical University, Kaohsiung, Taiwan; 11 Department of Medical Laboratory Science and Biotechnology, Kaohsiung Medical University, Kaohsiung, Taiwan; 12 AIDS Clinical Center, National Center for Global Health and Medicine, Tokyo, Japan; 13 Center for AIDS Research, Kumamoto University, Kumamoto, Japan; National and Kapodistrian University of Athens, GREECE

## Abstract

The usefulness of ultra-deep pyrosequencing (UDPS) for the diagnosis of HIV-1 drug resistance (DR) remains to be determined. Previously, we reported an explosive outbreak of HIV-1 circulating recombinant form (CRF) 07_BC among injection drug users (IDUs) in Taiwan in 2004. The goal of this study was to characterize the DR of CRF07_BC strains using different assays including UDPS. Seven CRF07_BC isolates including 4 from early epidemic (collected in 2004–2005) and 3 from late epidemic (collected in 2008) were obtained from treatment-naïve patient’s peripheral blood mononuclear cells. Viral RNA was extracted directly from patient’s plasma or from cultural supernatant and the pol sequences were determined using RT-PCR sequencing or UDPS. For comparison, phenotypic drug susceptibility assay using MAGIC-5 cells (in-house phenotypic assay) and Antivirogram were performed. In-house phenotypic assay showed that all the early epidemic and none of the late epidemic CRF07_BC isolates were resistant to most protease inhibitors (PIs) (4.4–47.3 fold). Neither genotypic assay nor Antivirogram detected any DR mutations. UDPS showed that early epidemic isolates contained 0.01–0.08% of PI DR major mutations. Furthermore, the combinations of major and accessory PI DR mutations significantly correlated with the phenotypic DR. The in-house phenotypic assay is superior to other conventional phenotypic assays in the detection of DR variants with a frequency as low as 0.01%.

## Introduction

Combination antiretroviral therapy (cART), also known as highly active antiretroviral therapy (HAART) can decrease the morbidity and mortality of HIV-1/AIDS patients [1–3]. However, the emergence of HIV-1 drug resistance (DR) may lead to cART failure [4, 5]. Therefore, detection of DR viruses is important for clinical management of HIV-1/AIDS. Two assays have been designed for the detection of HIV-1 DR: genotypic and phenotypic assays [6]. Genotypic assay uses direct PCR amplification of the HIV-1 pol region followed by Sanger sequencing (also called bulk sequencing). It is widely used in the clinical laboratory diagnosis of HIV-1 DR since it is less expensive and has a short processing time [6]. However, the results of these assays do not always represent the clinical outcome because resistance is predicted by mutations that had been previously observed [7]. In addition, the specimens need to contain at least 20% of the DR quasispecies or variants [8, 9]. In contrast, phenotypic assays measure HIV-1 viral replication in cells cultured in different drug concentrations. There are two types of phenotypic assays: commercially available phenotypic assays generate chimeric viruses by homologous recombination of PCR-derived sequences and then culture with cells in different drug concentrations [10, 11] and in-house phenotypic assay use peripheral blood mononuclear cells (PBMCs) to isolate HIV-1 and then incubate them in target cells (MAGIC-5 cells) with different drug concentrations [12, 13]. It has been reported that phenotypic drug resistance using recombinant virus assay was limited to detect low-frequency viral quasispecies below than 50% [14]. However, there is no data on the sensitivity of the in-house phenotypic assay which uses primary isolates from the patients directly.

Compared with standard population sequencing, a number of ultrasensitive assays, including allele-specific PCR and deep sequencing, can detect mutations present at a far lower frequency [15–17]. Low-frequency variants containing non-nucleoside reverse transcriptase inhibitor (NNRTI) resistance mutations were associated with virologic failure in patients receiving first-line cART [18]. In addition, using allele-specific PCR, Rowley et al. demonstrated that low-frequency variants containing K103N and Y181C increased the risk of treatment failure of nevirapine [19]. One of the approaches is ultra-deep pyrosequencing (UDPS) which sequences millions of PCR amplicons, such as sequencing on the Roche 454 platform. However, few studies have been conducted to evaluate the usefulness of UDPS in the detection of low-frequency DR variants in clinical settings [18, 20–22].

In Taiwan, HIV-1 circulating recombinant form (CRF) 07_BC is one of the predominant strains in injection drug users (IDUs) [23, 24]. The risk factors associated with IDU infection and the virological characteristics of CRF07_BC have been well addressed in our previous study [24–28]. However, little is known about the characteristics of the DR profiles of treatment naïve patients infected with CRF07_BC. Previously we performed in-house phenotypic and genotypic assay to determine the DR profiles in two treatment naïve IDUs infected with CRF07_BC. In-house phenotypic assay [12] showed that one IDU who was an early seroconverter had phenotypic DR to PIs. However, no DR mutations were observed in the HIV-1 pol regions using genotypic assay. Therefore, we proposed that low-frequency of PI-resistant variants may exist in CRF07_BC infected patients that cannot be detected by genotypic assay but can be identified through in-house phenotypic assay.

## Materials and Methods

### Subjects

Seven CRF07_BC isolates including 4 from early epidemic (collected in 2004–2005) and 3 from late epidemic (collected in 2008) were obtained from treatment-naïve patient’s PBMCs. Demographic data was assessed through a self-administered questionnaire. PBMCs were collected for primary culture and HIV-1 subtyping. Blood plasma was collected for viral RNA extraction.

### Ethics statement

This study was approved by the Institutional Review Boards (IRB) of Kaohsiung Medical University Chung-Ho Memorial Hospital. Written informed consent was obtained from patients who agreed to participate in this study.

### HIV-1 subtyping and primary culture with PBMCs

Viral RNA was extracted from plasma using QIAmp viral RNA mini kits (Qiagen, Hilden, Germany). DNA sequencing was performed using a ABI PRISM 3700 DNA analyzer (Applied Biosystems, Foster City, USA). HIV-1 subtyping was determined using nested multiplex polymerase chain reaction (PCR) and phylogenetic tree analysis as described previously [26, 28]. Briefly, two sets of nested PCR were performed to determine the HIV-1 subtype. One set added three pairs of primers for subtype B, C and CRF01_AE. Subtype can be determined by the different size of PCR products. Another set of nested PCR was performed using CRF07_BC specific-primer pairs to discriminate CRF07_BC from subtype C. Virus production was performed by donor PBMCs co- culture with infected PBMCs. The culture procedures have been described elsewhere [29, 30].

### Virus stock titration

MAGIC-5 cells were counted and dispensed into a 96-well tissue culture plate at 4.5×10^3^ cells/well with growth medium. Medium was removed the next day, and the virus diluted by growth medium plus DEAE-dextran (20μg/ml) (Sigma, Saint Louis, USA) was added to each well. Infection by each virus was performed in triplicate wells. Cells were assayed for infection by staining for β-galactosidase expression at 48 hours post-infection. Culture medium was removed, and fixing solution (0.1% formaldehyde and 0.02% glutaraldehyde in PBS) was added to each well. The monolayer was fixed at room temperature for 5 min, and 100μl of staining solution (4mM potassium ferrocyanide, 4mM potassium ferricyanide, 2mM magnesium chloride, and 400μg/ml 5-bromo-4-chloro-3-indolyl-β-D-galactopyranoside [X-gal] in PBS) was added to each well. The number of blue-stained cells was counted and expressed as blue cell-forming units (BFUs).

### In-house phenotypic assay

Each clinical isolate was directly tested for drug susceptibility in triplicate using MAGIC-5 cells as described previously [12]. Briefly, each drug was prepared in four serial 10-fold dilutions with infection medium (growth medium supplemented with 20μg/ml of DEAE-Dextran). MAGIC-5 cells were counted and dispensed into a 96-well tissue culture plate at 4.5×10^3^ cells/well with growth medium. To determine the susceptibility to PIs, 400–600 BFU of virus isolates with or without serial-diluted drugs were added to wells. The cells were cultured for 4–5 days for multiple-round replication, and the supernatant was transferred to another 96-well tissue culture plate with MAGIC-5 cells. In this step, the virus was cultured in a drug-free medium, and the cells were fixed and stained as described above after 48 hours of drug-free culture. To determine the susceptibilities to nucleoside reverse transcriptase inhibitors (NRTIs) and NNRTIs, 100 BFU of virus isolates with or without serial-diluted drugs were added to wells, and the cells were fixed and stained after 48 hours of co-culture with virus and drugs. Blue infected cells were counted under an inverted microscope, and the 50% inhibitory concentration (IC_50_) of each drug was estimated from plots of the percentage of BFU reduction versus drug concentration. Each experiment used the NL4.3 HIV-1 strain as control. For comparison, an in vitro phenotypic resistance assay that measures the level of resistance of recombinant HIV-1 from plasma samples was performed using Antivirogram assay [11].

The NIH AIDS Research and Reference Reagent Program (Division of AIDS, National Institute of Allergy and Infectious Diseases [NIAID], NIH) provided the antiviral drugs used in this study: PIs, ritonavir (RTV), saquinavir (SQV), indinavir (IDV), nelfinavir (NFV), amprenavir (APV); NRTIs, zidovudine (AZT), lamivudine (3TC), stavudine (d4T), didanosine (ddI), zalcitabine (ddC), abacavir (ABC), tenofovir (TDF); NNRTIs, nevirapine (NVP), efavirenz (EFV).

In this study, all of the CRF07_BC isolates were cultured with MAGIC-5 cells containing different concentrations of RT inhibitors or PI inhibitors. Nine NRTIs, 2 NNRTIs and 5 PIs were used in the in-house phenotypic assay. DR was determined by comparing the fold increase of IC_50_ with NL4.3 control. Fold increase was interpreted and assigned a phenotypic resistance or susceptibility using the clinical cut off values (CCOs) or biological cut off values (BCOs). CCOs was defined as the clinical relevant breakpoints of treated patients with drug resistant HIV-1. BCOs was defined as the breakpoints of drug susceptibility range of wild-type virus in the in vitro susceptibility assay. If the fold difference between patient isolates and NL4.3 control was above the upper CCO value or BCO value, the isolates were defined as having DR. Drug susceptibility was defined as a fold increase below the lower CCO. Intermediate DR was a fold increase between the lower and upper CCOs. In addition, the fold increase interpretation used BCOs when CCOs were lacking. However, BCOs are not derived from data of clinical responses to ARV drugs and may lack clinical relevance [31–34].

### Genotypic assay

Viral RNA was reverse transcribed to cDNA using Tetro cDNA synthesis kit (Bioline, Taunton, USA) with a random hexamer. The HIV-1 pol gene was amplified by PCR using Blend Taq^plus^ kit (Toyobo, Osaka, Japan) with the first (F1849 and R3500) and nested primers (MAW26 and RT21) published previously [35]. Briefly, the first PCR reaction for HIV-1 pol region was performed using cDNA as template. The products of the first PCR process continued with nested PCR. Sequences of the pol region were compared to the consensus B sequence from the Stanford HIV DR Database (http://hivdb.stanford.edu/) to detect and estimate mutations of DR and susceptibility to PIs and reverse transcriptase inhibitors (RTIs).

### Ultra-Deep Pyrosequencing (UDPS)

Amplicon sequencing was performed according to the manufacturer’s instructions. PCR was done using Expand High Fidelity DNA polymerase (Roche Applied Science, Basel, Switzerland). The purified PCR products were used in direct population Sanger sequencing (ABI 3730, Applied Biosystems, Foster City, USA) and UDPS (Roche/454 GS Junior, Branford, USA). The PCR amplicons were sequenced by forward direction on the Roche 454 GS Junior platform. Equimolar pooling of the DNA molecular for each patient was performed and followed by emulsion PCR and pyrosequencing on a 454 GS Junior sequencer.

### UDPS sequences analysis and bioinformatics

Data cleaning was important to increase the quality of sequences for subsequent analysis. First, sequences from each run were separated into different multiple identifiers (MIDs), and then the data was cleaned by several steps. Sequences containing the following were discarded: 1) sequences that could not be separated by different MIDs, 2) sequences with a consecutive PHRED score of less than 20, 3) sequences with < 80% similarity to the corresponding Sanger sequence, 4) sequences containing ambiguous bases (Ns), 5) sequences with lengths shorter than 300 base pairs, and 6) sequences containing insertions, deletions and stop codons. In the remaining sequences, the proportion of mutations in the protease region was calculated (S1 Fig) (S1 Table). The strength of relationship between phenotypic DR and low frequency DR variants was estimated by a Pearson correlation coefficient. The difference was considered statistically significant when p< 0.05.

We have deposited our sequences data in the NIH short read archive (SRA). Data can be public accessed by theses accession numbers: SRS1830977 to SRS1830983.

## Results

### Patient demographics and clinical characteristics

A total of 7 male IDUs were recruited in this study. All of them were treatment naïve patients with CRF07_BC infection. The mean age of patients was 37.6 years (range, 28–51 years). Patients were separated into two groups defined as early epidemic (4 patients collected in 2004) and late epidemic (3 patients collected in 2008). Three of 4 early epidemic patients were early seroconverters (Table 1).

**Table 1:** Demographic and clinical data of HIV-1 infected patients.

Patient	Gender	Age	Year of diagnosis	Year of collection	CD4/μl	HBsAg	HCVAb	Detuned assay^a^
CRF07_BC								
Early epidemic								
TW_D38	M	37	Jun.2004	Nov.2004	325	-	+	Positive
TW_D53	M	38	Sep.2004	Nov.2004	364	-	+	Positive
TW_D83	M	28	Mar.2003	Dec.2004	360	-	+	NT
TW_D78	M	51	2004	Dec.2004	322	-	+	Positive
Late epidemic								
TW_D848	M	31	Dec.2006	Mar.2008	NT	-	+	NT
TW_D854	M	37	Nov.2004	Mar.2008	NT	-	+	NT
TW_D855	M	41	Sep.2005	Mar.2008	NT	+	+	NT

^a^ Determined by BED-CEIA.

NT, Not test.

### Early epidemic CRF07_BC isolates have phenotypic DR to most PIs using in-house phenotypic assay

The result of in-house phenotypic assay showed that all the early epidemic isolates had DR to most PIs (4.4- to 47.3-fold increase compared with CCO or BCO). For ritonavir (RTV), TW_D38, TW_D53 and TW_D83 had phenotypic DR (5.8-, 8.9- and 8.3-fold increase, respectively). All the early epidemic isolates had phenotypic DR to amprenavir (APV). For nelfinavir (NFV), TW_D38 and TW_D83 had phenotypic DR (27.4- and 47.3- fold increase, respectively). None of the late epidemic isolates had phenotypic DR to the PIs. However, two early epidemic isolates (TW_D53 and TW_D78) and one late epidemic isolate (TW_D855) had intermediate phenotypic DR to NFV (Table 2). Most early epidemic and late epidemic isolates did not have phenotypic DR to most RTIs. However, TW_D53 and TW_D78 had phenotypic DR to nevirapine (NVP) (9.77-fold increase) and tenofovir (TDF) (3.47-fold increase), respectively. TW_D38 had intermediate phenotypic DR to atazanavir (AZT) (2.9-fold increase) (Table 3). In addition, the results of Antivirogram showed that none of the CRF07_BC isolates had phenotypic DR to PIs and RTIs.

**Table 2:** Fold increase of IC_50_ of Taiwanese CRF07_BC for various protease inhibitors.

		Fold increase (IC_50_ [μM]±S.D) ^a^						
		Patients from early epidemic				Patients from late epidemic		
PIs	Lower CCO—Upper CCO	TW_D38	TW_D53	TW_D83	TW_D78	TW_D848	TW_D854	TW_D855
RTV	3.5*	5.8 (0.04±0.014)	8.9 (0.06±0.011)	8.3 (0.06±0.001)	1.7 (0.06±0.004)	1.2 (0.01±0.001)	0.8 (0.03±0.019)	1.1 (0.05±0.014)
APV	2.5*	4.4 (0.03±0.020)	5.4 (0.03±0.029)	5.5 (0.03±0.023)	6.1 (0.04±0.003)	0.2 (0.00±0.00)	1.0 (0.55±0.034)	0.8 (0.03±0.009)
NFV	1.2–9.4	27.4 (0.02±0.012)	3.6 (0.00±0.004)	47.3 (0.04±0.006)	5.0 (0.03±0.020)	1.4 (0.01±0.001)	1.2 (0.01±0.001)	2.3 (0.02±0.010)
SQV	3.1–22.6	1.2 (0.01±0.000)	1.0 (0.01±0.000)	1.2 (0.01±0.000)	1.2 (0.01±0.001)	0.7 (0.00±0.00)	0.7 (0.00±0.003)	1.0 (0.01±0.000)
IDV	2.3–27.2	1.1 (0.01±0.001)	0.9 (0.00±0.001)	1.0 (0.01±0.001)	1.4 (0.01±0.001)	1.1 (0.01±00.00)	1.0 (0.01±0.00)	1.5 (0.01±0.00)

RTV, ritonavir; SQV, saquinavir; IDV, indinavir; NFV, nelfinavir; APV, amprenavir.

^a^ Fold increase was calculated by dividing the IC_50_ of each drug by the mean IC_50_ for NL4.3 in each assay.

* These drugs have only one biological cut-off value (for in vitro susceptibility).

**Table 3:** Fold increase of IC_50_ of Taiwanese CRF07_BC for various reverse transcriptase inhibitors.

		Fold increase (IC_50_ [μM]±S.D) ^a^						
		Patients from early epidemic				Patients from late epidemic		
RTIs	Lower CCO—Upper CCO	TW_D38	TW_D53	TW_D83	TW_D78	TW_D848	TW_D854	TW_D855
NRTI								
AZT	1.5–11.4	2.90 (0.04±0.01)	1.92 (0.03±0.02)	0.91 (0.02±0.01)	1.38 (0.55±0.10)	0.85 (0.16±0.14)	0.92 (0.23±0.25)	1.82 (0.45±0.36)
3TC	1.2–4.6	1.24 (1.09±0.45)	1.31 (1.14±1.13)	0.79 (0.69±0.18)	0.38 (0.68±0.18)	0.66 (1.52±1.14)	1 (5.23±2.88)	0.79 (4.12±2.95)
d4T	1.0–2.3	0.59 (3.11±2.35)	0.92 (5.85±1.87)	0.87 (4.94±1.61)	1.10 (6.22±1.14)	0.17 (1.51±0.69)	0.76 (4.55±2.05)	0.61 (3.65±1.32)
ddI	0.9–2.6	0.69 (3.87±1.32)	1.69 (6.03±4.45)	0.95 (5.72±0.60)	1.16 (5.19±0.29)	0.52 (2.42±1.48)	0.66 (5.58±1.14)	0.72 (6.06±0.67)
ddC	3.5*	0.78 (0.78±0.06)	1.21 (0.85±0.23)	0.73 (0.73±0.06)	0.91 (0.64±0.02)	0.29 (0.59±0.06)	0.26 (0.77±0.04)	0.27 (0.78±0.28)
ABC	0.9–3.5	0.51 (3.11±1.04)	1.03 (6.25±0.77)	0.79 (4.79±1.09)	1.24 (6.66±0.48)	0.55 (2.47±1.21)	0.75 (5.19±1.22)	0.51 (3.52±0.29)
TDF	1.0–2.3	0.90 (0.04±0.01)	1.89 (0.08±0.01)	1.09 (0.05±0.01)	3.47 (0.04±0.01)	0.79 (0.008±0.00)	0.75 (0.02±0.01)	0.51 (0.01±0.01)
NNRTI								
NVP	6.0*	0.99 (0.08±0.03)	9.77 (0.79±0.07)	0.93 (0.08±0.01)	4.91 (0.36±0.09)	0.71 (0.14±0.06)	0.23 (0.07±0.01)	0.02 (0.07±0.01)
EFV	3.3*	1.04 (0.003±0.00)	1.44 (0.005±0.00)	1.57 (0.005±0.00)	1.42 (0.005±0.00)	0.78 (0.005±0.00)	0.91 (0.006±0.00)	0.68 (0.005±0.00)

AZT, zidovudine; 3TC, lamivudine; d4T, stavudine; ddI, didanosine; ddC, zalcitabine; ABC, abacavir; TDF, tenofovir; NVP, nevirapine; EFV, efavirenz

^a^ Fold increase was calculated by dividing the IC_50_ of each drug by the mean IC_50_ for NL4.3 in each assay.

* These drugs have only one biological cut-off value (for in vitro susceptibility).

The results of genotypic assay showed that none of the patients had major DR mutations in the protease and reverse transcriptase regions. In the protease region, the following accessory mutations including E35D, M36I, R41K, D60E, L63P and I93L were detected in all the patients. I13M mutation occurred in TW_D38 and TW_D53. N37H and N37P occurred in TW_D78 and TW_D854, respectively. I64M and V77I occurred in TW_D848 and TW_D854, respectively. However, most of the mutations mentioned above were defined as amino acid polymorphic mutations according to the Stanford HIV-1 DR database. D60E and L63P are polymorphic mutations and more common in patients receiving PIs (Table 4 and S2 Table).

**Table 4:** The amino acid changes in the protease region in Taiwanese CRF07_BC strains.

	Amino acid residue									
Patient	I13	E35	M36	N37	R41	D60	L63	I64	V77	I93
CRF07_BC										
CN54	-	D	I	-	K	E	P	-	-	L
Early epidemic										
TW_D38	M	D	I	-	K	E	P	-	-	L
TW_D53	M	D	I	-	K	E	P	-	-	L
TW_D78	-	D	I	H	K	-	P	-	-	L
TW_D83	-	D	I	-	K	E	P	-	-	L
Late epidemic										
TW_D848	-	D	I	-	K	E	P	M	-	L
TW_D854	-	D	I	P	K	E	P	-	I	L
TW_D855	-	D	I	-	K	E	P	-	-	L

Amino acids identical to consensus B sequence (top) were indicated with dashes. CN54 was the prototypic CRF07_BC strain from mainland China.

Taken together, all the early epidemic isolates have phenotypic DR to most PIs. While none of them have DR observed by both genotypic and Antivirogram assays. We speculate that the phenotypic PI DR of early epidemic isolates was caused by low-frequency variants containing PI major DR mutations. Therefore, we performed UDPS to detect the low-frequency variants of the CRF07_BC isolates.

### Low-frequency PI DR variants were positively associated with phenotypic PI DR

The low-frequency variants of CRF07_BC isolates were detected by UDPS. After different steps of quality control, the remaining sequences were analyzed for correlation with phenotypic PI DR. We analyzed the correlation between phenotypic PI DR and the sequences containing major mutations along or concurrent with major mutations and accessory mutations in the protease region. As shown in Table 5, the low-frequency PI DR variants associated with phenotypic PI DR occurred at a frequency of 0.01 to 0.08. For RTV, the low-frequency PI DR variants harboring the major mutation I54S concurrent with accessory mutations M36I, L63P and I93L had positive correlation with phenotypic PI DR (R = 0.87, p = 0.01). For APV, the low-frequency PI DR variants harboring the major mutation I54V had positive correlation with phenotypic PI DR (R = 0.91, p< 0.01). For NFV, the following mutations had positive correlation with phenotypic PI DR: D30N + G73S (R = 0.85, p = 0.01), I54T + A71V (R = 0.85, p = 0.01), I54M + M36I + L63P + I93L (R = 0.85, p< 0.01), I54L + M36V (R = 0.91, p< 0.01) and V82F + M36I + L63P + I93L (R = 0.99, p< 0.01).

**Table 5:** Correlation between the phenotypic drug susceptibility and low frequency variants in Taiwanese CRF07_BC early epidemic isolates.

			Fold increase^a^ / Percentage of major or major + accessory mutations			
PIs	Correlation (R)	P value	TW_D38	TW_D53	TW_D83	TW_D78
RTV						
** I54S** + M36I + L63P + I93L	0.87	0.01	5.8 / 0	8.9 / 0.02	8.3 / 0.01	-
APV						
** I54V**	0.91	< 0.01	4.4 / 0.04	5.4 / 0.05	5.5 / 0.06	6.1 / 0.08
NFV						
** D30N** + G73S	0.85	0.01	-	-	47.3 / 0.01	-
** I54T** + A71V	0.85	0.01	-	-	47.3 / 0.01	-
** I54M** + M36I + L63P + I93L	0.85	< 0.01	-	-	47.3 / 0.01	-
** I54L** + M36V	0.91	< 0.01	27.4 / 0.01	-	47.3 / 0.01	-
** V82F** + M36I + L63P + I93L	0.99	< 0.01	27.4 / 0.01	-	47.3 / 0.01	-

^a^ Fold increase was calculated by dividing the IC_50_ of each drug by the mean IC_50_ for NL43 in phenotypic drug susceptibility assay.

Bold indicates PI major drug resistance mutations.

In summary, we collected 7 CRF07_BC isolates including 4 from early epidemic (collected in 2004–2005) and 3 from late epidemic (collected in 2008). The in-house phenotypic assay showed that all of the early epidemic and none of the late epidemic CRF07_BC isolates were resistant to most PIs (4.4–47.3 fold). Neither genotypic assay nor Antivirogram detected any DR mutations. UDPS showed that early epidemic isolates contained 0.01–0.08% of PI DR major mutations. Furthermore, the combinations of major and accessory PI DR mutations significantly correlated with the phenotypic PI DR.

## Discussion

In this study, in-house phenotypic assay showed that early epidemic CRF07_BC isolates from treatment naïve patients had phenotypic DR to most PIs, while genotypic assay and Antivirogram showed that none of them had DR mutations in the HIV-1 protease region. UDPS showed that early epidemic isolates contained 0.01–0.08% of PI DR major mutations. Furthermore, the combinations of major and accessory PI DR mutations significantly correlated with phenotypic PI DR.

In this study, the low-frequency PI DR variants could be detected with the in-house phenotypic assay but not with the other phenotypic assays. The difference between Antivirogram assay and in-house phenotypic assay is the method of virus preparation. The former uses a chimeric virus generated by homologous recombination of RT-PR PCR-derived sequences, which is then cultured with cells and different drug concentrations [10, 11]. The latter uses PBMCs co-culture to isolate HIV-1 and is then incubated in MAGIC-5 cells with different drug concentrations [12, 13]. It has been demonstrated that the DR mutations can impair HIV-1 fitness [36–40]. However, several studies have shown that mutations in HIV-1 gag region can compensate the fitness loss from the DR mutations [41, 42]. Other studies have shown that strong selection and loss of certain genotypes occur on virus cultivation [43–45]. The chimeric virus contains only the RT-PR sequence of HIV-1. Therefore, the Antivirogram assay was unable to detect the low-frequency resistance variants, as they could be influenced by partial loss of low-frequency variants due to reduced fitness of viruses containing the DR mutations and decreased number of low-frequency resistance variants in the population. In contrast, we isolated the patient’s virus by PBMCs co-culture. These culture isolates can provide more accurate results than the chimeric virus. In addition, the detection of low-frequency variants has been used in clinical settings. For instance, low-frequency variants have been shown to predict the treatment failure to NNRTIs [46]. Another study found that use of Roche 454 UDPS can be a potentially better predictor of maraviroc response than the original phenotypic test [47].

Based on phenotype effects, mutations that cause resistance to PIs can be classified as major mutations and accessory mutations. Major mutations are frequently associated with a several fold increase in resistance to one or more PIs. Accessory mutations, on the other hand, do not cause a great increase in resistance to PIs. If a major mutation occurs in a genetic background containing accessory mutations, resistance to PIs may be augmented. In this study, low-frequency variants containing major PI DR mutations (at positions 30, 54 or 82) and accessory mutations (at positions 36, 63, 71, 73 or 93) significantly correlated with the phenotypic PI DR (Table 5). Several studies showed that, for the major PI DR mutations observed by UDPS, I54V/A/S/T/L/M mutations reduced the drug susceptibility to most PIs and most occurred in patients receiving multiple PIs [48–51]. It has been reported that D30N is exclusively selected by NFV and confers resistance to this drug [52]. For the accessory mutations observed using UDPS, M36I is a consensus mutation frequently occurring in non-B subtypes and particularly in PI-experienced patients [50, 53]. M36I can increase the replication capacity when combined with PI major resistance mutations. In addition, I36 introduced into HIV-1 subtype B strain showed a higher replication capacity in both the absence and presence of PIs [54]. Moreover, the position 36 polymorphism in the HIV-1 protease region had different drug susceptibility to PIs and the effect depended on the viral subtype [55]. L63P and I93L are mutations frequently seen in non-B subtypes and PI-treated patients. L63P mutation compensated the impairment of fitness caused by the major DR mutation [56]. I93L mutation showed had a hypersusceptibility to lopinavir in the presence of a subtype C protease backbone [57]. Another study showed that the I93L mutation was associated with resistance to RTV [58]. This is the first study to use UDPS to analyze the correlation between the combinations with major DR and accessory mutations with the phenotypic DR to PIs in Taiwanese CRF07_BC infected treatment naïve patients. Further study is needed to determine the proportion of CRF07_BC infected patients containing low-frequency PI DR variants and their clinical treatment outcome.

In this study, we could still detect the transmitted low frequency PI DR variants in early epidemic patients after CRF07_BC was transmitted to Taiwan in 2002. It is well known that most DR mutations reduce the replication capacity of HIV-1, and the transmitted DR mutations can rapidly revert to wild type in the absence of drug pressure [59–62]. However, a recent study showed that CRF07_BC has slow replication kinetics and lower viral load than subtype B [25]. We speculate that the lower replication capacity of CRF07_BC may affect the DR mutations reverting to wild type. Moreover, we speculate that the transmission of PI DR variants among Taiwanese IDUs infected with CRF07_BC originated from China. The Chinese government initiated free cART for HIV-1/AIDS patients in 2002 [63]. However, from our understanding, there was a clinical trial of HIV vaccine conducted in Yunnan Province, where IDUs recruited, and if they were tested positive, they will be offered one year free treatment since they were not eligible for the participation (personal communication). Therefore, it is possible that PI DR emerged in the IDUs in Yunnan Province was transmitted to Taiwanese IDUs in 2002 and these DR variants were detected in early epidemic patients recruited in 2004–2005. These low-frequency variants can contribute to phenotypic DR to PIs. In addition, the Chinese government initiated free treatment of RTIs to HIV-1 infected patients in 2002. However, all the patients recruited in this study did not have DR resistance to RTIs using in-house phenotypic, Antiviragram and genotypic assays. Due to the vital role of reverse transcriptase in the early phase of HIV-1 replication. We speculated that DR mutation occurs in reverse transcriptase region can cause substantial reductions in viral fitness. Therefore, low-frequency variants containing DR mutation in reverse transcriptase can decrease dramatically. However, further studies are needed to compare the impact on viral fitness of DR mutation in reverse transcriptase and protease regions.

Previous studies showed that most of the CRF07_BC were CCR5-tropic virus and had non-syncytium-inducing (NSI) phenotype using genotypic and phenotypic assays [25, 64]. However, several studies have demonstrated that T-cell-tropic (X4) and macrophage-tropic (R5) viruses can effectively produce using MAGIC-5 cells. Therefore, both R5-tropic and X4-tropic viruses can using MAGIC-5 cells to determine the HIV-1 drug susceptibility to PIs and RTIs [12, 13].

There are several limitations to this study. The patients recruited in this study were not followed up to determine the dynamics of the low-frequency variants. A small number of patients was recruited into this study. Despite the limitations, this study was able to demonstrate that both in-house phenotypic assay and UDPS can detect low-frequency DR variants as low as 0.01%.

In conclusion, this study showed that early epidemic patients infected with CRF07_BC had phenotypic DR to PIs. Moreover, the combinations of major and accessory PI DR mutations significantly correlated with the phenotypic DR. In addition, our in-house phenotypic assay correlated well with UDPS results and both methods can detect the low-frequency variants of HIV-1 quasispecies.

## Supporting Information

S1 Fig The bioinformatics pipeline that processed sequences generated by UDPS. First, reads were separated by different MIDs and then combined fna and qual to fastq. Second, reads containing PHRED score smaller than 20, read length smaller than 350 base pairs and ambiguous bases (Ns) were discarded. Third, reads similarity with corresponding viral genome greater than 80% were retained for further processing. Fourth, reads containing insertions, deletions and stop codon were discarded. The variations of nucleotides and amino acids every position were calculated in the remaining reads.
(TIF)

S1 Table Number of sequences discarded from isolates by different filtering steps. (DOCX)

S2 Table The amino acid changes in the reverse transcriptase region in Taiwanese CRF07_BC strains. (DOCX)
