# Supplementary figures and images for: Characterization of the Drug Resistance Profiles of Patients Infected with CRF07_BC Using Phenotypic Assay and Ultra-Deep Pyrosequencing
Source: PLoS One. 2017 Jan 20;12(1):e0170420. doi: 10.1371/journal.pone.0170420 (PMC5249062; doi:10.1371/journal.pone.0170420)

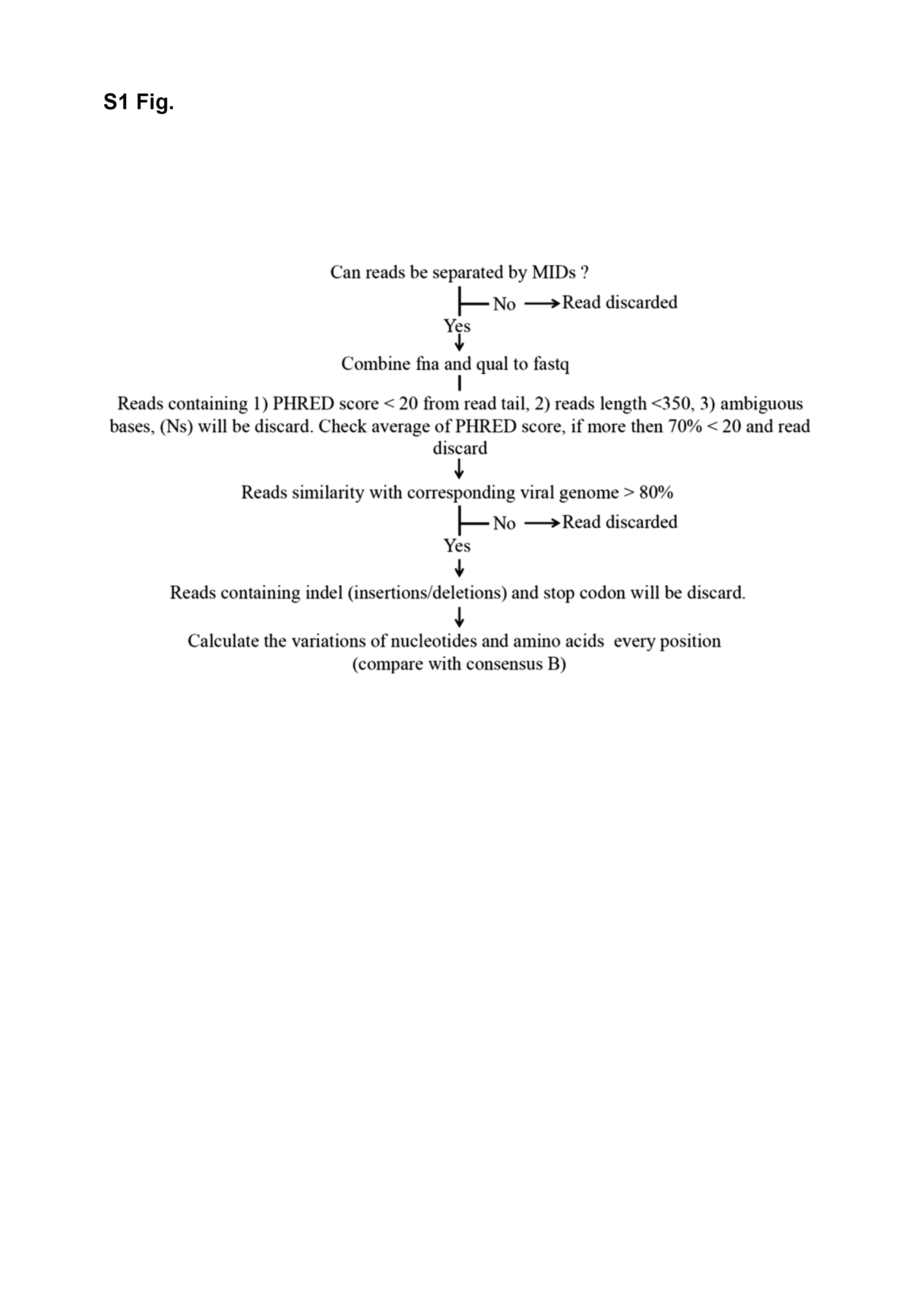

Supplement: S1 Fig — First, reads were separated by different MIDs and then combined fna and qual to fastq. Second, reads containing PHRED score smaller than 20, read length smaller than 350 base pairs and ambiguous bases (Ns) were discarded. Third, reads similarity with corresponding viral genome greater than 80% were retained for further processing. Fourth, reads containing insertions, deletions and stop codon were discarded. The variations of nucleotides and amino acids every position were calculated in the remaining reads. (TIF) [file pone.0170420.s001.tif]
