# Supplementary material for: Characterization of the Drug Resistance Profiles of Patients Infected with CRF07_BC Using Phenotypic Assay and Ultra-Deep Pyrosequencing
Source: PLoS One. 2017 Jan 20;12(1):e0170420. doi: 10.1371/journal.pone.0170420 (PMC5249062; doi:10.1371/journal.pone.0170420)
Supplement: S1 Table — (DOCX) [file pone.0170420.s002.docx]

| S1 Table. Number of sequences discarded from isolates by different filtering steps. | | | | | | | | |
| --- | --- | --- | --- | --- | --- | --- | --- | --- |
|  | No. of discarded | | | | | | | |
|  | Patients from early epidemic  (Number of total reads) | | | |  | Patients from late epidemic  (Number of total reads) | | |
| Steps of sequences filtering | TW_D38  N=33,145 | TW_D53  N=34,261 | TW_D83  N=30,941 | TW_D78  N=20,372 |  | TW_D848  N=27,772 | TW_D854  N=28,041 | TW_D855  N=27,404 |
| 1 | 199  (0.6%) | 216  (0.6%) | 230  (0.7%) | 109  (0.5%) |  | 361  (1.3%) | 548  (1.9%) | 592  (2.2%) |
| 2 | 161  (0.5%) | 294  (0.9%) | 219  (0.7%) | 141  (0.7%) |  | 701  (2.6%) | 813  (2.9%) | 1,131  (4.2%) |
| 3 | 7  (0.02%) | 6  (0.02%) | 118  (0.4%) | 14  (0.1%) |  | 24  (0.1%) | 394  (1.5%) | 241  (0.9%) |
| 4 | 12,766  (38.9%) | 13,789  (40.9%) | 11,504  (37.9%) | 8,469  (42.1%) |  | 16,163  (60.6%) | 14,855  (56.5%) | 14,879  (58.5%) |
| No. of final sequences | 20,012 | 19,956 | 18,870 | 11,639 |  | 10,523 | 11,431 | 10,561 |
| N, number of input reads.  Steps, 1) Trimming reads from tail (PHRED score < 20) and reads length <350. 2) Filter reads containing ambiguous bases, (Ns) and check average of PHRED score (if more than 70% < 20). 3) Filter reads with similarity with corresponding viral genome < 80%. 4) Filter reads that containing indel (insertions/deletions) and stop codon. | | | | | | | | |
