# Supplementary material for: Characterization of the Drug Resistance Profiles of Patients Infected with CRF07_BC Using Phenotypic Assay and Ultra-Deep Pyrosequencing
Source: PLoS One. 2017 Jan 20;12(1):e0170420. doi: 10.1371/journal.pone.0170420 (PMC5249062; doi:10.1371/journal.pone.0170420)
Supplement: S2 Table — (DOCX) [file pone.0170420.s003.docx]

| S2 Table. The amino acid changes in the reverse transcriptase region in Taiwanese CRF07_BC strains. | | | | | | | | | | | | | | | | | | | | | | | | | | | | | | | | | | | | | | | | |
| --- | --- | --- | --- | --- | --- | --- | --- | --- | --- | --- | --- | --- | --- | --- | --- | --- | --- | --- | --- | --- | --- | --- | --- | --- | --- | --- | --- | --- | --- | --- | --- | --- | --- | --- | --- | --- | --- | --- | --- | --- |
|  |  | | Amino acid residue | | | | | | | | | | | | | | | | | | | | | | | | | | | | | | | | | | | | | |
| Patient | E  6 | V  8 | | K  1  1 | K  3  2 | V  3  5 | E  3  6 | T  3  9 | S  4  8 | K  4  9 | V  6  0 | R  8  3 | K  1  0  3 | D  1  2  1 | K  1  2  2 | S  1  3  4 | I  1  3  5 | I  1  4  2 | R  1  4  3 | I  1  5  9 | K  1  6  6 | E  1  6  9 | R  1  7  2 | I  2  0  2 | E  2  0  4 | Q  2  0  7 | R  2  1  1 | F  2  1  4 | V  2  4  5 | S  2  5  1 | A  2  7  2 | V  2  7  6 | K  2  7  7 | T  2  8  6 | E  2  9  1 | V  2  9  2 | I  2  9  3 | E  2  9  7 | E  2  9  8 | K  3  1  1 |
| CRF07_BC |  |  | |  |  |  |  |  |  |  |  |  |  |  |  |  |  |  |  |  |  |  |  |  |  |  |  |  |  |  |  |  |  |  |  |  |  |  |  |  |
| CN54 | - | - | | - | - | T | A | D | T | - | I | - | - | Y | E | - | R | - | - | - | - | - | - | - | - | - | - | - | Q | - | P | - | R | A | D | I | - | - | - | - |
| Early epidemic |  |  | |  |  |  |  |  |  |  |  |  |  |  |  |  |  |  |  |  |  |  |  |  |  |  |  |  |  |  |  |  |  |  |  |  |  |  |  |  |
| TW_D38 | - | - | | - | - | T | A | D | T | R | I | - | - | Y | E | - | - | L | G | - | - | D | - | - | - | - | - | - | Q | - | P | - | R | A | D | I | V | - | - | R |
| TW_D53 | - | - | | - | - | T | A | D | T | R | I | - | - | Y | E | R | - | - | - | - | - | D | - | - | - | - | - | - | Q | - | P | - | R | A | D | I | V | - | - | R |
| TW_D78 | - | I | | - | - | T | A | D | T | R | I | - | - | Y | E | - | T | - | - | - | - | D | - | - | - | - | - | - | Q | - | P | - | R | A | D | I | V | - | - | - |
| TW_D83 | - | - | | - | E | T | A | D | T | R | I | - | - | Y | E | - | R | - | - | - | - | D | - | - | - | - | - | - | Q | - | P | - | R | A | D | I | V | - | - | - |
| Late epidemic |  |  | |  |  |  |  |  |  |  |  |  |  |  |  |  |  |  |  |  |  |  |  |  |  |  |  |  |  |  |  |  |  |  |  |  |  |  |  |  |
| TW_D848 | - | - | | R | - | T | A | D | T | R | I | - | - | Y | E | - | - | - | - | - | - | D | - | - | - | - | - | - | Q | - | P | - | R | A | D | I | V | - | - | R |
| TW_D854 | - | I | | - | - | T | A | D | T | R | I | - | - | Y | E | - | K | - | - | Y | - | D | K | - | - | - | - | - | Q | - | P | - | R | A | D | I | V | - | - | - |
| TW_D855 | - | I | | - | - | T | - | E | T | R | I | - | - | Y | E | - | R | - | - | - | - | D | - | - | - | - | - | - | Q | - | P | - | R | A | D | I | V | - | - | R |
| Amino acids identical to consensus B sequence (top) are indicated with dashes. CN54 was the prototypic CRF07_BC strain from mainland China. | | | | | | | | | | | | | | | | | | | | | | | | | | | | | | | | | | | | | | | | |
